# Hypothermia After Cardiac Arrest in Large Animals (HACA-LA): Study protocol of a randomized controlled experimental trial

**DOI:** 10.1016/j.resplu.2024.100704

**Published:** 2024-06-29

**Authors:** Olof Persson, Anna Valerianova, Jan Bělohlávek, Tobias Cronberg, Niklas Nielsen, Elisabet Englund, Mikuláš Mlček, Hans Friberg

**Affiliations:** a Department of Clinical Sciences, Anaesthesiology and Intensive Care, Lund University, Lund, Sweden; b Department of Intensive and Perioperative Care, Skåne University Hospital, Lund, Sweden; c Third Department of Internal Medicine, General University Hospital, Charles University, Prague, Czech Republic; d Institute of Physiology, First Faculty of Medicine, Charles University, Prague, Czech Republic; e Second Department of Medicine – Department of Cardiovascular Medicine, First Faculty of Medicine, Charles University and General University Hospital, Prague, Czech Republic; f Department of Clinical Sciences, Neurology, Lund University, Lund, Sweden; g Department of Neurology, Skåne University Hospital, Lund, Sweden; h Department of Clinical Sciences, Pathology, Lund University, Lund, Sweden; i Department of Genetics, Pathology and Molecular Diagnostics, Skåne University Hospital, Lund, Sweden; j Department of Intensive and Perioperative Care, Skåne University Hospital, Malmö, Sweden

**Keywords:** Cardiac arrest, Hypothermia, Temperature control, Functional outcome, Swine

## Abstract

**Background:**

Induced hypothermia post-cardiac arrest is neuroprotective in animal experiments, but few high-quality studies have been performed in larger animals with human-like brains. The neuroprotective effect of postischemic hypothermia has recently been questioned in human trials. Our aim is to investigate whether hypothermia post-cardiac arrest confers a benefit compared to normothermia in large adult animals. Our hypothesis is that induced hypothermia post cardiac arrest is neuroprotective and that the effect diminishes when delayed two hours*.*

**Methods:**

Adult female pigs were anesthetized, mechanically ventilated and kept at baseline parameters including normothermia (38 °C). All animals were subjected to ten minutes of cardiac arrest (no-flow) by induced ventricular fibrillation, followed by four minutes of cardiopulmonary resuscitation with mechanical compressions, prior to the first countershock. Animals with sustained return of spontaneous circulation (systolic blood pressure >60 mmHg for ten minutes) within fifteen minutes from start of life support were included and randomized to three groups; immediate or delayed (2 h) intravenous cooling, both targeting 33 °C, or intravenously controlled normothermia (38 °C). Temperature control was applied for thirty hours including cooling time, temperature at target and controlled rewarming (0.5 °C/h). Animals were extubated and kept alive for seven days. The primary outcome measure is histological brain injury on day seven. Secondary outcomes include neurological and neurocognitive recovery, and the trajectory of biomarkers of brain injury.

**Conclusion:**

High-quality animal experiments in clinically relevant large animal models are necessary to close the gap of knowledge regarding neuroprotective effects of induced hypothermia after cardiac arrest.

**Trial registration:**Preclinicaltrials.eu (PCTE0000272), published 2021-11-03.

## Introduction

Out-of-hospital cardiac arrest (OHCA) is a major health issue with an annual incidence of 67–170 per 100,000 inhabitants.^1^ Survival rates at hospital discharge are low, less than 10% survive, most of whom with a good neurological outcome.^1^

The brain has a high oxygen demand and is vulnerable to global ischemia, hence brain injury is a major cause of mortality and morbidity in cardiac arrest patients.^2^, ^3^ The immediate cessation of oxygen delivery causes anaerobic metabolism and intracellular acidosis due to accumulation of metabolites.^4^ A sudden lack of adenosine triphosphate (ATP) with malfunctioning of ATP-dependent ion pumps lead to impaired ion homeostasis.^5^ Increased intracellular levels of sodium generates cytotoxic edema.^6^ Further, intracellular overload of calcium plays a key role in a secondary injury cascade with the release of neurotoxic transmitter substances, activation of lytic enzymes and mitochondrial dysfunction with the forming of reactive oxygen species (ROS) and release of deleterious proteins.^7^, ^8^, ^9^ This cascade is accompanied by further pathological processes including inflammation, disruption of the blood–brain barrier, regional perfusion defects and dysfunctional autoregulation.^10^, ^11^, ^12^ The overall consequence is an instant as well as a delayed type of cell death, the latter allowing for a potential therapeutic window in post-resuscitation care.^13^, ^14^

Post-ischemic hypothermia decreases cerebral metabolism by 5–10% per 1 °C, which reduces oxygen demand and mitigates anaerobic conditions. Mitochondrial dysfunction, release of harmful neurotransmitters, ROS-production and inflammation are all reduced by hypothermia.^15^, ^16^, ^17^ Early experimental studies showed neuroprotective effects from hypothermia during and after cardiac arrest.^18^ In 2002, two separate clinical trials investigating post-ischemic hypothermia, targeting 32–34 °C for 12–24 h, showed improved outcomes.^19^, ^20^ The results from these two small trials changed clinical practice worldwide and induced hypothermia after cardiac arrest was recommended by international guidelines.^21^, ^22^ In 2013, and in 2021, two large randomized trials showed no benefit of targeting a temperature of 33 °C compared to either 36 °C or avoiding fever (<37.8 °C) post-cardiac arrest.^23^, ^24^ The neuroprotective effect of post-ischemic hypothermia in clinical practice was thus questioned. A recent systematic review and meta-analysis has shown no benefit and current international guidelines do not recommend its use.^25^, ^26^ A return to the experimental setting is thus essential to explore whether a therapeutic effect of induced hypothermia post-cardiac arrest exists and, if so, to define a potential therapeutic window.

Recent reviews of induced hypothermia post-cardiac arrest in the experimental setting show a beneficial effect, however, few high-quality studies have been conducted in large animals with gyrencephalic human-like brains.^27^, ^28^ A key to solving the translational gap is to conduct high-quality experiments in relevant animal models. We therefore aim to test the following hypotheses in large animals:

• Induced hypothermia after cardiac arrest is neuroprotective
• Early induction of hypothermia improves neuroprotection compared to delayed hypothermia

## Methods

The HACA-LA-trial study protocol has been approved by the Institutional Animal Expert Committee at First Faculty of Medicine, Charles University, and experiments were performed at the experimental laboratory, Institute of Physiology, First Faculty of Medicine, Charles University, Prague, Czech Republic, in accordance with Act No. 246/1992 Coll., on the protection of animals against cruelty and EU Directive 2010/63/EU**.** The study will be reported according to ARRIVE and Utstein style guidelines.^29^, ^30^

### Experimental animals

Female swine (*Sus scrofa domestica*, Large White × Landrace crossbreed), five to six months of age with a body weight of 50–75 kg, were used. Animals had free access to food and water and were kept in separate boxes. A local breeder provided, non-specific-pathogen-free animals, which arrived two weeks prior to cardiac arrest for acclimatization. Medications to relieve pain and distress were given on demand, all medications are specified in Appendix A1*.*

### Preparation and monitoring

After overnight fasting, general anesthesia was induced by intramuscular administration of midazolam (0.3 mg/kg) and ketamine hydrochloride (15–20 mg/kg). Following intravenous (IV) boluses of propofol (2 mg/kg) and morphine (0.1–0.2 mg/kg), an endotracheal intubation was performed. General anesthesia was maintained by infusions of propofol (4–20 mg/kg/h) and remifentanil (0.6–1.2 μg/kg/min). The depth of anesthesia was adjusted according to demand. During the intervention, rocuronium (1.0–3.0 mg/kg/h) was infused to prevent shivering.

Volume control ventilation (Hamilton G5, Hamilton Medical AG, Switzerland) with 8 ml/kg tidal volume was used. Oxygen fraction, positive end-expiratory pressure and respiratory rate was adjusted to achieve an oxygen saturation of 94–98% and end-tidal CO_2_ pressure of 4.6–6.0 kPa. Basal fluid replacement was given with Ringer’s Acetate (2 ml/kg/h) after an initial fluid bolus to achieve a central venous pressure of 5–6 mmHg for baseline. Femoral and jugular veins and arteries were used for multiple sheath insertions with Seldinger technique. A heparin IV bolus of 5000 IU was given and followed by continuous IV infusion 35–70 U/kg/h to maintain an activated clotting time of 180–220 s (Hemochron Jr Signature Plus Microcoagulation System, ITC, NJ, USA). Urinary and vaginal temperature probes were placed and an endovascular temperature control catheter (Quattro©, Zoll Medical, CA, USA) was used. Cardiac output was monitored with a Swan-Ganz catheter (Vigilance II, Edward Lifesciences, USA). Near-infrared spectroscopy oximetry (INVOS, Medtronic, Minneapolis, MN, USA) was used to monitor tissue perfusion of the brain, limbs and body. Electrocardiographic parameters, heart rate, invasive blood pressures, pulse oximetry, capnometry, and invasive central venous oxygen saturation was continuously recorded (Monitor Life Scope TR, Nihon Kohden MU-651RK, Japan). A four-channel electroencephalogram (EEG) was recorded during the intervention (Nicolet Endevour CR, Natus Medical, WI, USA).

### Cardiac arrest

Animals were kept at baseline parameters, specified in Appendix A2, including normothermia (38 ± 0.2 °C) fifteen minutes prior to cardiac arrest. Ventricular fibrillation (VF) was induced with rapid pacing through a wire placed in the right ventricle. At cardiac arrest the endovascular temperature management device was inactivated, all infusions stopped, and the ventilator disconnected.

All animals were subjected to ten minutes of no-flow (NF) from VF, followed by four minutes of low-flow (LF). Basic Life support (BLS), with mechanical compressions provided by the LUCAS™ Chest Compression System (Physio-Control/Jolife AB, Sweden) in combination with manual bag-valve ventilation, with an oxygen flow of 15 l/min, at a ratio of 30:2. After three minutes of BLS, a bolus dose of adrenaline IV (1 mg) was given to improve perfusion pressures at first countershock.^31^ Further Advanced life support (ALS) including two stacked shocks of 200 J (Zoll X series, Zoll Medical, CA, USA) was performed every two minutes of persistent VF, starting four minutes from the onset of VF. Adrenaline boluses were repeated one minute before shocks throughout CPR. A bolus dose of amiodarone IV (300 mg) was given in combination with adrenaline one minute before the second round of countershocks and a second bolus dose of amiodarone IV (150 mg) was given before the third round of countershocks. Full details of the resuscitation are specified in Appendix A3.

**Fig. 1:**
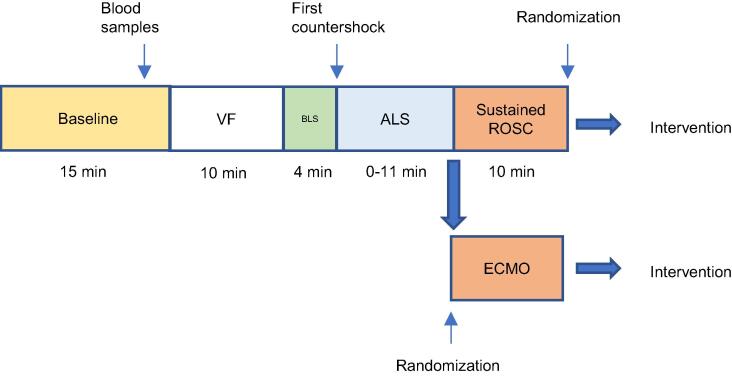
Cardiac arrest timeline.

Stable ROSC was defined as ten minutes of sustained systolic blood pressure >60 mmHg, vasopressors and inotropes were allowed to maintain the blood pressure. Animals with Stable ROSC were eligible for randomization. If ROSC was not acquired within fifteen minutes of total CPR-time the animals were entered into a separate study including initiation of ECMO, described in Appendix A4. If re-arrest occurred prior to randomization, CPR was applied again for a total maximum of fifteen minutes, including previous LF-time and stable ROSC counted from the latest conversion (Fig. 1).

### Interventions

Mechanical ventilation was reconnected at ROSC and inspired oxygen fraction is set at 100% in all animals for 30 min for equal initial oxygen exposure. Sedation was maintained until the end of the intervention period. Intensive care treatment was provided to achieve the physiological goals specified in Appendix A5.

Animals achieving stable ROSC were randomized into three arms: Early hypothermia group, Delayed hypothermia group and Normothermia group, as specified below:

The Early hypothermia group received immediate cooling via a rapid infusion (10 min) of cold (4 °C) Ringer’s Actetate (30 ml/kg) and simultaneous induction of temperature control with the endovascular cooling device (Coolguard™, Zoll Medical, CA, USA) set at maximum cooling rate targeting 33 ± 0.2 °C. Temperature control was maintained for eighteen hours, followed by controlled rewarming for ten hours at a rate of 0.5 °C/h. The animals were kept sedated with temperature control at 38 ± 0.2 °C for two more hours to compensate for the delay time in the Delayed hypothermia group.

The Delayed hypothermia group received temperature control at 38 ± 0.2 °C for a two-hour period before initiation of cooling. After two hours, cooling with a target of 33 ± 0.2 °C was initiated and conducted in the same manner as in the Early hypothermia group.

**Fig. 2:**
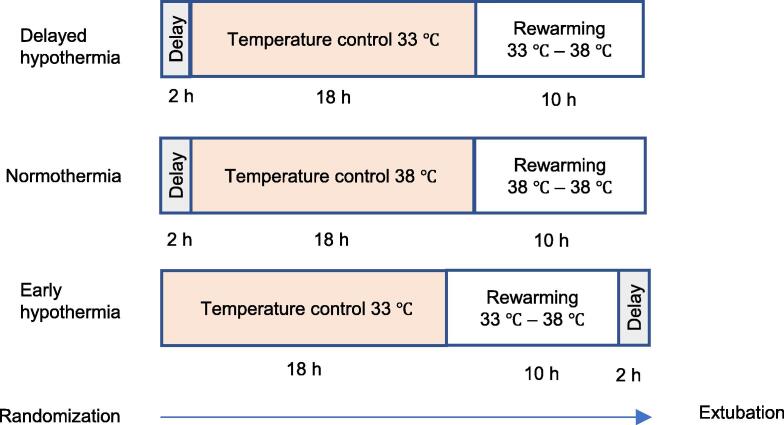
Intervention timeline.

The Normothermia group was kept at 38 ± 0.2 °C during the entire intervention period, at the lower end of normal temperature range in swine (38–39 °C).^32^ These animals received a rapid fluid bolus after a delay period of two hours to align with the Delayed hypothermia group timewise, but the fluid was at room temperature (25 °C). The total intervention period was thirty hours for all three groups (Fig. 2).

### Post-intervention

After the intervention, animals were weaned from ventilator and extubated. Starting from CA, a total experimental time of seven days was planned. Daily follow-up examinations were conducted. Basal physiological parameters including heart rate, respiratory rate and temperature were recorded.

Animals were euthanized with potassium chloride (2 mEq/kg), combined with a high dose of general anesthesia on day 7. The brains were removed after euthanasia and immediately preserved in formaldehyde prior to histopathologic examination. Criteria for preemptive euthanasia are specified in Appendix A6.

### Outcome measures

#### Primary outcome

Brain injury will be assessed according to a modified Histology Damage Score, a modification from the Histology damage score in pigs published by Högler et al.^33^ Modifications are based on a human study published by Björklund et al., later expanded by Haglund et al.^13^, ^14^

Brains were immersed in formaldehyde solution 4%, then cut for selection of regions to embed in paraffin. Subsequently the paraffin blocks were sectioned at 4 µm and stained with Hematoxylin-eosin. Two independent pathologists, blinded to allocation group, will assess the brain damage in tissue sections with a light microscope. Four brain regions will be assessed: Neocortex, Thalamus, Hippocampus and Cerebellum. A score between 0 and 4 will be given based on severity (0 = no damage, 4 worst damage) for three different injury types: edema (weighting factor 1), eosinophilic neuronal death (weighting factor 2) and pyknosis (weighting factor 2). The score for each injury type will be multiplied by its weighting factor and added up to a total sum for each region. All four regions’ total sum will be divided by the number of regions to attain a final modified Histology Damage score between 0 and 20. Animals not surviving until the end of the experiment automatically will get a final maximal modified Histology Damage score of 21.

#### Secondary outcomes

Brain injury according to modified Histology Damage score in animals that did not survive the entire pre-planned follow-up.

Neurological examination according to a Neurological Deficit Score by Sipos et al., performed one day prior to the intervention and once daily after the intervention, the protocol is specified in Appendix A7.^34^

A model for neurocognitive testing by Fries et al. was performed.^35^ Animals were trained to find and open food troughs on time, once daily during five days before cardiac arrest. The procedure was repeated during four days of the follow-up period.

**Fig. 3:**
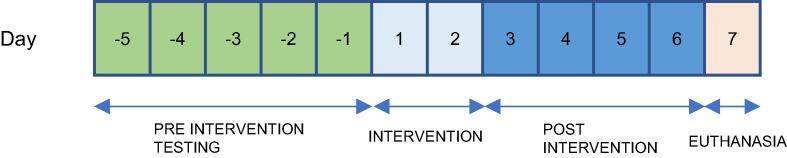
Experimental timeline overview.

Biomarkers of brain injury were collected at baseline (5 min prior to cardiac arrest), 2 h, 12 h, 24 h and 48 h post randomization, and at the end of follow-up period. We plan to analyze neurofilament light chain, neuron-specific enolase, glial fibrillary acidic protein and tau protein (Fig. 3).

### Inclusion and exclusion criteria

#### Inclusion

• Baseline parameters acquired according to protocol
• Cardiac arrest and stable ROSC according to protocol

#### Exclusion

• Re-arrest after randomization not responding to two cycles of CPR (4 min)
• Sustained inability to maintain adequate perfusion pressure and oxygenation (>60 min) during the intervention period

### Sample size

An a priori sample size calculation has been performed. We estimated a mean modified Histology Damage score of 16 and 12 in the control group (Normothermia group) and main intervention group (Delayed hypothermia group) respectively with a standard deviation of 2.6 in both groups, based on previous studies.^33^, ^36^, ^37^ Our calculation suggested that we needed 10 animals per arm to reach a Power of 80% and a Significance level of 2.5%. We assumed that the third arm (Early hypothermia group) would need to be of the same size which leads to a total of 30 animals. Our power calculation was based on a *t*-test and in order to compensate for the lower power achieved with Mann-Whitney U tests, one additional animal in each intervention arm was added, totaling 33 animals. This addition was made after the initial registration on preclinicaltrials.eu, prior to any data analysis.

### Randomization

The interventions were randomized with sealed envelopes at stable ROSC. Randomization was performed in blocks of three, each block containing the three arms. A reason for using blocked randomization was to spread the interventions evenly over time in order to avoid seasonal impact from infectious diseases.^38^, ^39^, ^40^

### Blinding

The modified Histology Damage score assessment, biomarker analyses and all statistical analysis will be performed by investigators blinded to the allocation groups. The investigators conducting the experiments were not blinded to allocation due to practical difficulties. The neurological examination and neurocognitive testing assessments were not blinded for the same reason, however, the assessments were video-filmed and will be validated by a blinded assessor.

### Statistical methods

Data from all animals will be analyzed and published. The three groups will be compared with an overall test as well as pairwise comparisons between groups. Since the scale is ordinal for the primary outcome the non-parametric Kruskal-Wallis and Mann-Whitney U tests are used. Pairwise comparisons will be corrected with Bonferroni correction which lowers the significance level to 2.5%. Non-parametric tests will be used for secondary outcomes as well since sample sizes are small.

## Discussion

We present an experimental protocol to investigate the therapeutic potential for induced hypothermia after cardiac arrest in large animals. Our aim is to investigate the neuroprotective effect of induced hypothermia in a clinically relevant animal model, i.e. with a two-hour delay after ROSC, and compare with controlled normothermia. Our aim is also to investigate whether immediate hypothermia after ROSC improves the neuroprotective effect compared to delayed hypothermia.

In a publication by Russel et al., three fundamental principles to optimize animal experiments are presented; Replacement, Reduction and Refinement.^41^ These principles highlight our responsibility to effectively conduct animal experiments. As to our study, the lack of high-quality trials in gyrencephalic animals regarding induced hypothermia after cardiac arrest motivates our choice of study population. Second, an a priori sample size calculation aims to minimize the number of animals and yet maintain the prerequisites for a robust data set. Third, we intend to maximize the potential benefit of the study animals by introducing a separate ECMO study for animals not achieving ROSC. The injury model with no-flow time of ten minutes has been selected in order to induce a severe enough injury with maintained ROSC rates, based on previous studies and our own pilot experiments.^33^, ^42^ All procedures have been designed in adherence with relevant guidelines in order to optimize quality and minimize pain and discomfort.^29^, ^43^.

The optimal dose of temperature control in terms of temperature goal and maintenance time is unknown. A recent meta-analysis addressing animal studies by Arrich et al. suggests an improved beneficial effect with lower temperatures, but surprisingly and in contrast to previous findings, a diminished effect by longer durations.^28^ Our model, with a delta drop of 5 °C compared to controlled normothermia for eighteen hours including cooling time, offers a clinically feasible model with a potential to improve the evidence regarding temperature control in large human-like animals. The possible therapeutic window for initiation of temperature control also needs further investigation. In our study design, the delayed hypothermia strategy mimics a transport plus *in-hospital* cooling scenario, while the early hypothermia strategy mimics a *preclinical* cooling scenario that continues in-hospital. The choice to use controlled normothermia as a control, in contrast to a laissez-faire approach regarding temperature, is based on results showing clear patterns of spontaneous *hyperthermia* in pigs after cardiac arrest in the experimental setting.^36^, ^44^ The pattern was also evident in our pilot experiments. This is in contrast to humans where core body temperature typically is reduced early after cardiac arrest with mean admission temperatures of approximately 35.3C in large trials.^23^, ^24^

Our follow-up model with repeated assessments of neurological function and neurocognition is meant to mimic the clinical routine post-cardiac arrest. The protocol thus states that weaning of sedation should be performed soon after the intervention, allowing for awakening and extubation.

### Strengths and limitations

The strengths of this study include the general design in a randomized controlled trial, planned in adherence with relevant and updated guidelines.^29^, ^30^ The prolonged follow-up period of seven days and the range of outcomes, histopathology, neurological function and neurocognition, offer opportunities to identify previously neglected (positive or negative) effects of hypothermia in the experimental setting. The risk of bias will be reduced by blinding of the assessors. The controlled laboratory environment and transparency regarding the results will offer an option to repeat all experiments. The use of continuous EEG and repeated sampling of brain injury biomarkers add strength to the model to quantify the ischemic brain injury. The laboratory setting with a healthy animal population and induced VF differs from the human OHCA population and will limit the generalizability of the results. Due to practical reasons, blinding of the interventions is not possible, and our blocked randomization also partly limits blinding, which increases the risk of bias.

## Conclusion

OHCA is a major health issue with dismal prognosis, and no evidence-based post-arrest interventions to improve outcomes are presently available.^2^ Induced hypothermia has showed neuroprotective effects in experimental settings, but results have not been possible to translate into the clinical setting. Well controlled experimental studies in human-like animals are required to close the research gap regarding neuroprotective effects of induced hypothermia after cardiac arrest.

## Status of the study

The original ethics application was approved in 2019 (Sweden). The ethics application for Prag, Czech Republic, was submitted in 2021 but a reply was never received (due to the pandemic), why a new ethics application was submitted 2023-11-27 and approved 2024-03-21. Inclusion of animals was finalized in October 2022, no brain histopathology (primary outcome) has been assessed and no unblinded data analysis has occurred (June 2024).

## Funding

This work was supported by The Swedish Heart Lung Foundation; Hans-Gabriel and Alice Trolle-Wachtmeisters Foundation for Medical Research. The authors are solely responsible for the design and conduction of this study, the analyzes and future publications of results.

## Ethics approval

Sweden, reference: Malmö – Lunds djurförsöksetiska nämnd: Dnr 5.8.18-16160/2019. Date of approval: 2019-12-18.

Czech Republic, reference: Department of education, Czech Republic, ref no: MSMT-31012/2023. Date of approval: 2024-03-21.

## CRediT authorship contribution statement

**Olof Persson:** Conceptualization, Investigation, Methodology, Project administration, Visualization, Writing – original draft. **Anna Valerianova:** Conceptualization, Investigation, Methodology, Writing – review & editing. **Jan Bělohlávek:** Conceptualization, Methodology, Writing – review & editing. **Tobias Cronberg:** Conceptualization, Funding acquisition, Methodology, Writing – review & editing. **Niklas Nielsen:** Conceptualization, Funding acquisition, Methodology, Writing – review & editing. **Elisabet Englund:** Conceptualization, Funding acquisition, Investigation, Methodology, Writing – review & editing. **Mikuláš Mlček:** Conceptualization, Investigation, Methodology, Resources, Writing – review & editing. **Hans Friberg:** Conceptualization, Funding acquisition, Methodology, Project administration, Supervision, Writing – review & editing.

## Declaration of competing interest

The authors declare that they have no known competing financial interests or personal relationships that could have appeared to influence the work reported in this paper.
