# Supplementary material for: Hypothermia After Cardiac Arrest in Large Animals (HACA-LA): Study protocol of a randomized controlled experimental trial
Source: Resusc Plus. 2024 Jun 29;19:100704. doi: 10.1016/j.resplu.2024.100704 (PMC11261465; doi:10.1016/j.resplu.2024.100704)
Supplement: Supplementary Appendix [file mmc1.docx]

# APPENDIX A

**A1 – Medications:**

**Premedication**:

| **Drug** | **Dose**  **bolus** | **Dose infusion** | **Route** | **Concentration** | **Indication** | **Other** |
| --- | --- | --- | --- | --- | --- | --- |
| ketamine | 15–20 mg/kg | x | IM/SC | 100 mg/ml | Premedication |  |
| midazolam | 0,3 mg/kg | x | IM/SC | 5 mg/ml | Premedication |  |

**Anesthesia:**

| **Drug** | **Dose**  **bolus** | **Dose infusion** | **Route** | **Concentration** | **Indication** | **Other** |
| --- | --- | --- | --- | --- | --- | --- |
| propofol | 1–4 mg/kg | 4–20 mg/kg/h | IV | 10 mg/ml | Induction, Sedation, Anticonvulsant | Infusion titration in steps of 1 mg/kg/h |
| remifentanil | x | 0,6–1,2 mcg/kg/min | IV | 100 mcg/ml | Sedation, Analgesia | Infusion titration in steps of 0,1 mcg/kg/min |
| rocuronium | 1 mg/kg | 1,0–3,0 mg/kg/h | IV | 10 mg/ml | Muscle relaxation | Infusion titration in steps of 0,5 mg/kg/h |
| midazolam | 0,3 mg/kg | x | IV | 5 mg /ml | Anticonvulsant, sedation | Bolus if convulsions /distress |

**Other medications:**

| **Drug** | **Dose**  **bolus** | **Dose infusion** | **Route** | **Concentration** | | **Indication** | **Other** |
| --- | --- | --- | --- | --- | --- | --- | --- |
| norepinephrine | 10–20 mcg | 0.01–1 mcg/kg/min | IV | 40 mcg/ml | | Vasopressor demand |  |
| epinephrine | 100–200 mcg | 0,01–2 mcg/kg/min | IV | 80 mcg/ml | | Vasopressor + Inotropy demand |  |
| amiodarone | 150/300 mg | x | IV | 50 mg/ml | | Arrythmia |  |
| magnesium sulfate | 10 mmol | x | IV |  | | Arrythmia | In 100 ml Saline 0,9% over 30 min |
| lidocaine | 1 mg/kg | 1 mg/kg/h | IV | 10 mg/ml | | Arrythmia |  |
| heparin | 5000 IU | 35/70 IU/kg/h | IV | 1 IU/ml | Anticoagulation | | Pre arrest: 70 IU/kg/h,  Post arrest: 35 IU/kg/h |
| furosemide | 5–20 mg | x | IV | 10 mg/ml | Fluid overload | |  |
| atropine | 0,5 mg | x | IV | 0,5 mg/ml | Bradycardia | |  |
| sodium bicarbonate | 100 ml | x | IV | 8,4% | Base deficit >6 | |  |
| epinephrine | 1 mg | x | Inhale | 1mg/ml | Airway Obstruction | | Solved in 4 ml of saline  (repeat on demand every 15 min) |

**Routine medications:**

| **Drug** | **Start** | **End** | **Dose (route)** | **Interval** | **Other** |
| --- | --- | --- | --- | --- | --- |
| cefazolin | Day 1 (08.00) prior to instrumentation | Day 3 (48 h post randomization) | 1 g (IV) | 8 h |  |
| paracetamol | Day 1 (11.00) at Randomization | Day 3 (48 h post randomization) | 1g (IV) | 6 h | To prevent fever |
| meloxicam | Day 2, 1 h prior to extubation | Day 7 | 0,4 mg/kg (Oral/SC) | Once daily | Oral when awake and able to eat |
| morphine | Day 2, 1 h prior to extubation | Day2, 20.00 | 10 mg (IV/SC) | 2 single bolus doses | Additional boluses on demand for pain/ distress |
| midazolam | x | x | 0,3 mg/kg (IV/SC) | x | On demand seizures/distress |

# **A2 – Baseline parameters:**

| **Parameters:** | **Values:** |
| --- | --- |
| Tidal volume (TV) | 8 ml/kg |
| Respiratory rate (RR) | 10-30/ min (lowest value to achieve target ET CO2 below) |
| Oxygen saturation (Sat) | > 98% |
| Positive end expiratory pressure (PEEP) | 5 cm H20 |
| End tidal carbon dioxide (ET CO2) | 35-40 mmHg |
| Mean arterial pressure (MAP) | 65-85 mmHg |
| Heart rate (HR) | SR, 50-80 bpm |
| Central venous pressure (CVP) | 5-6 mmHg |
| Cardiac output (CO) | >4 l/min |
| Temperature - bladder | 38 ± 0.2 °C |

#

| **Resuscitation protocol** | |
| --- | --- |
| 00:00 min | 30 chest compressions with LUCAS (100 min^-1^) and 2 manual ventilations with bag valve mask & 15 L/min oxygen flow (compressions discontinued during ventilations). |
| 03:00 min | Administer Adrenaline IV (1 mg) |
| 03:45 min | Pre-charge defibrillator to 200 J. |
| 04:00 min | 1st rhythm check and 1-2 shocks with rhythm check in between shocks and delivered if VF/pVT. Continue LUCAS with manual ventilations if unsuccessful shocks. |
| 05:00 min | Administer Adrenaline IV (1 mg). and add one dose of Amiodarone IV (300 mg) if the previous rhythm was shockable. |
| 05:45 min | Pre-charge defibrillator to 200 J. |
| 06:00 min | 2nd rhythm check and 1-2 shocks with rhythm check in between shocks and delivered if VF/pVT. Continue LUCAS with manual ventilations if unsuccessful shocks. |
| 07:00 min | Administer Adrenaline IV (1 mg) and add one half dose of Amiodarone IV (150 mg) if the previous rhythm was shockable. |
| 07:45 min | Pre-charge defibrillator to 200 J. |
| 08:00 min | 3rd rhythm check and 1-2 shocks with rhythm check in between shocks and delivered if VF/pVT. Continue LUCAS with manual ventilations if unsuccessful shocks. |
| 09:00 min | Administer Adrenaline IV (1 mg). |
| 09:45 min | Pre-charge defibrillator to 200 J. |
| 10:00 min | 4th rhythm check and 1-2 shocks with rhythm check in between shocks and delivered if VF/pVT. Continue LUCAS with manual ventilations if unsuccessful shocks. |
| 11:00 min | Administer Adrenaline IV (1 mg). |
| 11:45 min | Pre-charge defibrillator to 200 J. |
| 12:00 min | 5th rhythm check and 1-2 shocks with rhythm check between shocks and delivered if VF/pVT. Continue LUCAS with manual ventilations if unsuccessful shocks. |
| 13:00 min | Administer Adrenaline IV (1 mg). |
| 13.45 min | Pre-charge defibrillator to 200 J. |
| 14:00 min | 6th rhythm check and 1-2 shocks with rhythm check between shocks and delivered if VF/pVT. Continue ALS if unsuccessful shocks. |
| 15:00 min | If no ROSC: Not included in main trial and entered into the ECMO-trial. Start mechanical ventilation with 100% oxygen and 0 PEEP with same tidal volumes and RR as before CA and switch to continuous LUCAS compressions (100 min^-1^). Continue Adrenaline (1 mg) every 2 minutes until ECMO in place. |
| If PEA/ASYSTOLE at any time: Continued ALS-algorithm with rhythm checked every 2 minutes. Adrenaline given according to table. | |

**A3 – Resuscitation protocol:**

**A4 – ECMO study:**

Animals in the separate ECMO study were randomized to either normothermia or early hypothermia. Temperature control was achieved with ECMO. The temperature control regimens mimic the treatment arms in the main study regarding intervention time and targeted temperatures. Animals were not allowed to wake up, due to the risk of bleeding, hence all animals in this study were euthanized at the end of the intervention. Biomarkers and histopathology samples was collected and we aim to analyze these outcomes in a similar way as the main study.

**A5 – Intensive care treatment goals:**

| **Parameter** | **Goal** |
| --- | --- |
| Arterial partial pressure carbon dioxide (PaCO_2_) | 4.6-6.0 kPa = 35-45 mmHg |
| Arterial oxygen saturation (SaO_2_) | 94-98 % |
| Mean arterial pressure (MAP) | 65-85 mmHg |
| Mixed venous saturation (SvO_2_) | > 50 % |
| Cardiac output (CO) | > 4L/min |
| Central venous pressure (CVP) | 5-6 mmHg |
| Urinary output | >0,5 ml/kg/h |
| Activated clotting time (ACT) | 180–220 s |

|  | |
| --- | --- |
| **TEMPERATURE CONTROL (TC)** | |
| TC start-up | 0h / 2 h post ROSC |
| Target temperature | 33°C ± 0,2°C or 38,0 ±0,2 °C |
| Neuromuscular block | Bolus dose of 1 mg/kg at start of TC followed by infusion at 1.0-3.0 mg/kg/h. Start at lowest dose and titrate to effect on target = no shivering. Increase infusion in steps of 0,5 mg/kg/h and give bolus of 0,5 mg/kg with every increment. |
| **MAINTAINANCE TC** | |
| Duration | 18 hours (including induction) |
| Neuromuscular block continued | 1. – 3.0 mg/kg/hr rocuronium infusion. Titrated to effect on target, see above. |
| **REWARMING** | |
| Rewarming rate | 0.5°C/hr |
| Neuromuscular block continued | 1.0-3.0 mg/kg/hr rocuronium infusion Titrated to effect, see above. Stop infusion at T29 in all groups. |
| Normothermia | Active normothermia 38.0 ±0,2 °C |
| **POSITIONING**  Alternating between supine, prone, left lateral and right lateral every 4 h | |

**A6 – Criteria for pre-emptive euthanasia:**

- Severe irreversible hypoxia demanding reintubation
- Uncontrollable seizures/status epilepticus
- Uncontrollable pain
- Uncontrollable distress
- Uncontrollable hemorrhage
- Untreatable dehydration

**A7 – NDS (Sipos et al. 2008):**

| **Examination and score (0 = normal)** | **Instruction** |
| --- | --- |
| **MENTAL STATUS**  0 = normal  50 = not normal nor comatose  100 = comatose | 0 = normal:pig specific behaviour (highly interested in the environment, showing this by walking around, trying to examine everything with the snout and giving characteristic grunting sounds)  50 = not normal nor comatose  100 = comatose. Lateral position, possibly running movements, no reaction to any stimuli |
| **BREATHING**  0 = normal; 10 = abnormal; 20 = apnea | Abnormal: irregular, tachypnoea (>30/min), bradypnoe (<18/min) |
| **CRANIAL NERVES** |  |
| Pupillary light reflex: Right: 0/5; Left 0/5 | Shining a light in each eye and observing for pupillary constriction |
| Stabismus: Right: 0/5; Left 0/5 | Fixed deviation of globes |
| Nystagmus: Right: 0/5; Left 0/5 | Spontaneous nystagmus |
| Corneal reflex: Right: 0/5; Left 0/5 | Blink response to tactile stimulus of the cornea with a cotton swab |
| Ear reflex: Right: 0/5; Left 0/5 | Finger introduced into outer auditory canal, followed by twitch of the auricle |
| Menace reaction: 0/20 | Making a sudden, threatening gesture with the hand in the direction of one eye |
| Snout reflex: 0/20 | Pinching the nasal septum with two fingers |
| Swallowing reflex: 0/20 | Some water is to be administered laterally into the mouth with syringe |
| **MOTOR AND SENSORIC FUNCTION** |  |
| Muscle tonus: Foreleg 0/5; Hind leg 0/5 | Normal = neither spasticity nor paresis/paralysis |
| Positioning reaction: Foreleg 0/5; Hind leg 0/5 | Distal extremity is flexed, the pig should immediately return the foot to a normal position |
| Flexor reflex: Foreleg 0/5; Hind leg 0/5 | Flexor/withdrawal reflex, after pricking with a needle to interdigital cleft |
| Hopping reaction: Foreleg 0/15; Hind leg 0/15 | Shifting the animal to one side. First tries to oppose lateral pressure, then hops on the contralateral limb |
| Dorsal pressure reaction: Foreleg 0/15; Hind leg 0/15 | Pressure to the back is responded by contra-pressure |
| Panniculus reflex: 0/10 | Smooth paravertebral prickling of the skin with a needle |
| Perineal reflex: 0/10 | Smoothly prickling the perineum with a needle. Response is a contraction of the anal sphincter muscle and a ventralflexion of the tail |
| Standing: 0/15/30 | 0= self, 15 = with help, 30 = not able |
| Gait: 0/15/30 | 0= self, 15 = with help, 30 = not able |
|  |  |
| **Total:** | 0–400 points |
