# Supplementary material for: Hypothermia After Cardiac Arrest in Large Animals (HACA-LA): Study protocol of a randomized controlled experimental trial
Source: Resusc Plus. 2024 Jun 29;19:100704. doi: 10.1016/j.resplu.2024.100704 (PMC11261465; doi:10.1016/j.resplu.2024.100704)
Supplement: Supplementary Data 1 [file mmc2.pdf]

Pre-clinical registry ID	PCTE0000272
Date published (UTC)	2021-11-03 11:38:15 (submitted 2021-11-01 19:51:39)
All versions	2021-11-03 11:38:15
1. Title of the study	Hypothermia After Cardiac Arrest in Large Animals Trial
2. Acronym/short title	HACA-LA Trial
3. Contact details	Email the author
4. Study centre details	Lund University. Lund. Sweden<br><br>Charles University. Prague. Czech Republic
5. Sources of support	Grants
6. Start date	04/10/2021
7. Expected end date	04/04/2022
8. Study status	Active
Section 2. Study design	
9. Research field	Intensive Care Medicine
10. Research question/hypothesis	• Induced hypothermia after cardiac arrest (CA) and return of spontaneous circulation (ROSC) is neuroprotective • Early induction of hypothermia after ROSC improves neuroprotection compared to delayed hypothermia
11. In case of intervention, Intervention type	Other<br><br>Invasive whole body cooling post CA and ROSC
12. Study stage	Stage 2 – Confirmatory study (hypothesis testing)
13. Primary readout parameter	Histopathological brain damage will be assessed according to modified Histology damage score (mHDS) a modification from Histology damage score in pigs published by Högler et al. 2010 (1). Our modifications are based on a human study published by Björklund et al. 2014 (2). After

	euthanasia brains will be immersed in fixative solution and stained with Haematoxylin-eosin. A pathologist blinded to intervention will assess brain damage in tissue sections with light microscope. Four brain regions will be assessed: 1. Neocortex 2. Thalamus 3. Hippocampus 4. Cerebellum A score 0-4 for each of the three damage types below will be given based on severity (0 no damage, 4 worst): • Oedema (weighting factor 1) • Eosinophilic neuronal necrosis (weighting factor 2) • Pyknosis (weighting factor 2) The score for each damage type will be multiplied with its weighting factor and added up to a total sum between 0-20 for each region. All four regions total sum will be added up and divided by the total number of regions to attain compiled sum between 0-20 to constitute the final mHDS score. Animals not surviving until end of follow up period automatically get a final maximal score of 21 in primary outcome assessment.
14. Secondary readout parameter	• Daily Neurocognitive testing (NCT) pre and post intervention based on a model published by Fries et al. 2008 (3). • Daily neurlogical examination post intervention with Neurological deficit score (NDS) and Overall performance category (OPC) based on protocols published by Sipos et al. 2008 (4). • Serum biomarkers: neurofilament light chain (NFL), Neuron-specific enolase (NSE),glial fibrillary acidic protein (GFAP) and tau protein will be measured at the following timepoints: baseline pre-arrest, 2 h post randomization, 12 h post randomization, 24 h post randomization, 48 h post randomization and at end of follow-up period. • Brain damage according to mHDS (score 0-20) in animals not surviving until end of follow-up period.
15. Are animals exclusively used for this research question?	Yes
16. Species	Pig
17. Strain	Crossbreed Landrace/Large White Adult animals Weight 50-75 kg
18. Sex	Female
19. Provide the experimental design/protocol/ animal model	Adult pigs are anesthetized, kept at baseline parameters and subjected to 10 minutes of cardiac arrest by induced ventricular fibrillation. Basic life support is then commenced with mechanical compressions and manual ventilation provided for 4 minutes before countershocks are attempted. Animals who achieve stable ROSC, defined as sustained systolic blood pressure >60 mmHg for 10 min, within 15 minutes from start of life support are included and randomized into one of three arms: normothermia (goal 38 C with intravenous maintenance), delayed hypothermia (goal 33 C with intravenous cooling starting 2 hours post ROSC) or early hypothermia (goal 33 C with intravenous cooling starting at ROSC). Animals in all arms are anesthetized, muscle relaxed and supported by general intensive care with mechanical ventilation. Target temperature management is applied for 18 hours including cooling time. Animals in cooling arms are thereafter rewarmed at a rate of 0,5 C/h until reaching normothermia while the control group remains at normothermia. Intervention ends 30 hours post randomization in all arms and animals are weaned from intensive care and

	extubated. Follow-up is performed during 6 days after randomization including: biomarkers, daily neurological examination and neurocognitive testing. At the end of follow-up period animals are euthanized and brains are preserved for histopathological examination.
	Yes
20. Justify number of animals/Sample size calculation	• Power of 80% and a significance level of 2,5%. • Estimated mean mHDS of 16 and 12 in the control group and main intervention group (Delayed hypothermia) respectively and a standard deviation of 2,6 in both groups. • This means that we will need 10 animals in each group. To achieve balance in the trial we assume that the other intervention group (Early hypothermia) is of the same size which means that we in total need 30 animals.
21. Sum of animals	30
22. Study arms/groups	Type: intervention<br>Number: 10<br>Intervention: Delayed hypothermia<br><br>Type: intervention<br>Number: 10<br>Intervention: Early hypothermia<br><br>Type: control<br>Number: 10<br>Intervention: Normothermia
23. Randomisation	Are the animals randomly allocated to the experimental groups?<br><br>Yes<br><br>Method used - If randomisation is applied, please indicate the method used.<br><br>Shuffled blinded envelopes<br><br>Further details on randomisation - Provide further details on randomisation.<br><br>Block randomisation
24. Blinding	Are the investigators blinded at the time of intervention?<br><br>No

	Are the investigators blinded at the time of assessments of outcome(s)?<br><br>Yes<br><br>- Primary readout parameter: • mHDS assessment performed by an investigator fully blinded to intervention. - Secondary readout parameters: • Analysis and assessment of Serum biomarkers performed by an investigator fully blinded to intervention. • Assessment of NCT, NDS and OPC primarily performed by an investigator not blinded to intervention but filmed for secondary validation by an investigator fully blinded to intervention.
25. In case of an intervention, is it placebo-controlled?	Not applicable
26. Original animal ethics committee application or number of application	Malmö - Lunds djurförsöksetiska nämnd: Dnr 5.8.18-16160/2019
27. Additional information	
28. Link to data	1. https://doi.org/10.1016/j.resuscitation.2010.07.005 2. https://doi.org/10.1016/j.resuscitation.2013.11.022 3. https://doi.org/10.1097/CCM.0B013E3181653041 4. https://www.researchgate.net/publication/285667216_A_novel_highly_observer-independent_neurologic_examination_procedure_for_pigs_in_a_model_for_cardiac_arrest_resuscitation January 2008 Wiener Tierarztliche Monatsschrift 95(1):28-38
29. Statement of accuracy	Confirm
